# Evaluation of Adhesion Properties of Electrodeposited Copper Thin Films: Theoretical and Experimental Approach

**DOI:** 10.3390/ma18112480

**Published:** 2025-05-25

**Authors:** Ivana O. Mladenović, Jelena S. Lamovec, Dana G. Vasiljević-Radović, Rastko Vasilić, Vesna J. Radojević, Nebojša D. Nikolić

**Affiliations:** 1 Institute of Chemistry, Technology and Metallurgy, University of Belgrade, Njegoševa 12, 11000 Belgrade, Serbia; dana@nanosys.ihtm.bg.ac.rs; 2 University of Criminal Investigation and Police Studies, Cara Dušana 196, Zemun, 11000 Belgrade, Serbia; jelena.lamovec@kpu.edu.rs; 3 Faculty of Physics, University of Belgrade, Studentski Trg 12-16, 11000 Belgrade, Serbia; rastko.vasilic@ff.bg.ac.rs; 4 Faculty of Technology and Metallurgy, University of Belgrade, Karnegijeva 4, 11000 Belgrade, Serbia; vesnar@tmf.bg.ac.rs

**Keywords:** copper, electrodeposition, additives, film, microindentation, adhesion tests

## Abstract

The adhesion of copper thin films galvanostatically electrodeposited on Cu cathodes from electrolytes without or with the addition of various additives, such as chloride ions, polyethylene glycol 6000 (PEG 6000), and 3–mercapto–1–propanesulfonic acid, has been investigated. Morphological and structural analyses of synthesized films were performed using the SEM, AFM, and XRD methods, while the adhesion of the films was examined by applying the theoretical Chen–Gao (C–G) composite hardness model using results from Vickers microindentation, a bidirectional bending test, and a scratch-tape adhesion test. The morphologies of the films were either very smooth, with mirror-like brightness, obtained from the electrolyte containing all three additives, or microcrystalline, with different grain sizes, obtained from other electrolytes. The best adhesion was observed in the fine-grained film with numerous boundaries among grains, obtained with the addition of chloride ions and PEG 6000, while the mirror-bright film obtained with a combination of all three additives showed the worst adhesion. The boundaries among grains represented barriers that decreased the depth of penetration during microindentation and, consequently, increased the hardness and enhanced the adhesion of the film. The size of the grains—and hence, the number of grain boundaries—was regulated by the composition of the electrolytes achieved by the addition of additives. Good agreement was observed among the various methods used for the estimation of the adhesion properties of Cu films.

## 1. Introduction

Good adhesion of metal films to a substrate is a prerequisite for their application in various industrial branches. Generally, adhesion is governed by interatomic and intermolecular interactions at the interface between two surfaces [1,2]. From a thermodynamic point of view, the amount of energy required to create a free surface from bonded materials is the true work of adhesion at the interface [3], and this work can be measured using the energy of delamination of a film from a substrate.

Depending on the method of measurement, methods for evaluating the adhesion properties of materials can be categorized as quantitative/qualitative, destructive/non-destructive, and mechanical/non-mechanical [4]. Various methods—both theoretical and practical—have been reported for evaluating adhesion, such as nucleation (a theoretical, kinetics-based method), pull-off, centrifugal, ultrasonic, scotch tape, peel test, scribe, blister, abrasion, and lab shear tests [5]. The main techniques and test methods for measuring the adhesion of flexible thin films are the peel test, shear mode test, single and double cantilever beam method (SCB/DCB), tensile mode test, bidirectional bending test, and scratch test, where the choice of technique/method depends on the material combination of the film/substrate system and whether polymer or metal films are considered [6,7,8].

There are several criteria and factors that affect thin film adhesion: the process of substrate cleansing, film deposition rate, the thickness of the film, the substrate type, the temperature of the substrate, the purity of the source material, the evaporator’s pressure throughout the sputtering deposition process, and the purity of the electrolyte [9]. Surface adhesion can be altered using a variety of methods, such as altering the structure of the films or changing the substrate material surface chemically or physically [10]. When the substrate is prepared for deposition using physical surface treatment techniques (such as grinding with sandpaper), adhesion is enhanced due to roughening of the substrate. Surface roughening increases the substrate’s actual surface area and produces surface micro-porosity, which makes it easier for metal atoms to penetrate the surface and serve as nucleation sites for other metal atoms [11]. The creation of diverse chemical functional groups on the surface via suitable surface treatment techniques or deposition films with distinct functional groups is the primary cause of surface alteration in chemical adhesion promotion mechanisms [1,12].

Cu thin films are used in microelectronics as protective, sacrificial, or building elements, as well as in the production of various types of sensors, including both pressure and temperature sensors [13]. The excellent electrical properties of Cu ensure efficient performance of semiconductor devices such as diodes [14]. Thanks to their low resistivity and high reliability, Cu films are an ideal choice for interconnecting in integrated circuits (ICs). Also, Cu films help improve the efficiency of solar cells by providing a conductive layer that facilitates the flow of electricity [14]. It is clear that the realization of all listed components as well as devices requires good adhesion of the Cu film to the substrate.

Electrodeposition (ED) processes are a common way to produce Cu films of controllable surface morphology and structure, because both the morphology and structure of metal films can easily be regulated by choosing the appropriate regime and parameters of ED processes [15]. The type and composition of the electrolyte, temperature, mixing conditions, duration of the ED process (thickness), current density value used for ED, and cathode (substrate) type are the main parameters that strongly affect the morphology and structure of Cu films. Sulfate electrolytes based on copper sulfate and sulfuric acid are commonly used for Cu ED at the industrial scale [16,17]. Aside from sulfate electrolytes, rare uses of electrolytes based on cyanide, pyrophosphate, and chloride have also been reported [18]. Various additives can be included in electrolytes to modify the morphology and structure of electrodeposited copper. The most commonly used additives in Cu ED processes include thiourea, chlorides, polyethylene glycol (PEG), 3–mercapto–1–propanesulfonic acid (MPSA), gelatin, bis(3–sulfopropyl) disulfide (SPS), Janus Green B, etc. [19,20,21,22,23,24,25,26]. The addition of these additives, individually or in combination, such as chlorides, PEG 6000, and 3–mercapto–1–propanesulfonic acid (MPSA), strongly affects the morphology and structure of Cu films, enhancing smoothness and, moreover, leading to a mirror-like brightness [15,25].

These compounds, such as polyethylene glycol of various molecular weights (PEG) and 3–mercapto–1–propanesulfonic acid (MPSA), together with chloride ions, represent a new class of leveling/brightening additives, for which intense investigation and application in copper electrodeposition processes commenced in this century [27,28,29]. The addition of small amounts of chloride ions (up to several millimoles) in the sulfate electrolyte leads to the formation of an adsorbed CuCl layer at the electrode surface [30]. The formation of this adsorbed layer introduces an additional pathway in the Cu reduction process, simultaneously leading to a decrease in Cu electrodeposition overpotential. The addition of PEG alone in the sulfate electrolyte does not have any significant effect on the Cu reduction process, but when added together with chloride ions, it strongly inhibits or suppresses the reduction process [29]. In electrolytes with added PEG and chloride ions, the chloride ions act as bridging ligands, which facilitates PEG adsorption on the cathode surface by the formation of the PEG–Cu–Cl complex. As a result of the inhibition or suppression of the electrodeposition process, the increase in the electrodeposition overpotential relative to the additive-free electrolyte is observed. PEG and chloride ions achieve the leveling effect during the Cu electrodeposition process, while the brightening effect is realized by the addition of 3–mercapto–1–propanesulfonic acid. This additive, added in very small amounts, activates the electrodeposition process, and the model “local perforation” is proposed to explain the formation of mirror-bright films [21,22,24,31]. The MPSA molecules cause some kind of distortion of the formed PEG–Cu–Cl complex. Strongly binding to Cu atoms via their sulfur group, the MPSA molecules hinder full coverage of the Cu film by the formed complex, enabling the Cu (II) ions to penetrate into the growing film.

Although all listed parameters affect the adhesion of the formed films, the nature of the substrate (cathode) used in the ED process has the largest effect. The adhesion of a deposited layer on any substrate is frequently linked to the lattice mismatch at the interface of the two materials [32]. Electrochemically deposited metal films onto conductive substrates usually have excellent adhesion if the materials are the same and if the crystallographic lattices of the film and the substrate match. However, crystallographic coherency between the film and the substrate can degrade adhesion much more than imperfections in the film itself and at the interface (e.g., voids) [33,34]. For example, strong adhesion was observed at the interface of Ni film/Cu substrate, but poor adhesion was achieved in the system Cu film/Ni substrate, because hydrogen can diffuse more easily into the Ni substrate. Systems such as Ni film/Ag substrate, Ag film/Ni substrate, and Cu film/Ag substrate also have poor adhesion due to lattice incoherence, the appearance of voids at the film/substrate interface, and the occurrence of the so-called “anchor effect” [33].

Various methods/tests have been used for the examination of the adhesion properties of Cu thin films produced by the ED process. Tensile [35,36] and scratch [32] tests were used for the examination of the adhesion of Cu films electrodeposited directly onto a Si substrate. The destructive test method named the “scotch tape test” (ASTM D3359-02) is used for measuring the adhesion of copper films electrodeposited on a carbon steel substrate [8,32]. Assessment of the adhesion strength of mono- and multi-layered electrodeposited copper films produced without/with ultrasonic mixing of the electrolyte was investigated using a bidirectional bend method with a special bending fixture [7].

The aim of this work was to examine the adhesion of electrolytic copper films through the analysis of the influence of different additives added to a basic sulfate electrolyte. These parameters affect the morphology and structure of films, consequently influencing the adhesion properties of electrodeposited films. In order to easily perceive the effect of additives as well as their combination on the evaluation of adhesion properties, the same substrate (cathode) as the electrodeposited metal (Cu) was used in this study. Adhesion of Cu films was estimated by use of the theoretical Chen–Gao (C–G) composite hardness mathematical model [37,38,39,40,41,42,43] using experimental results obtained by Vickers microindentation and compared with two pure experimental methods (the bidirectional bend method and scotch tape test). Regarding the classification of methods used for evaluation of adhesion properties, they belong to different kinds: mechanical/non-destructive (microindentation adhesion test), mechanical/destructive (bidirectional bend method), and fast, descriptive but destructive methods (scotch tape test).

## 2. Materials and Methods

### 2.1. Electrodeposition Set-Up

Electrodeposition of Cu films on Cu cathodes was performed in constant galvanostatic (DC) mode, applying magnetic stirring (MS) (100 rpm, Heidolph MR 3001 K, Heidolph Instruments GmbH & Co. KG, Schwabach, Germany) at a temperature of 22.0 ± 0.5 °C in an open type of cell. The applied current density (*j*) value for the copper electrodeposition was 60 mA·cm^−2^, and the thickness of Cu films was 10 μm. The compositions of the electrolytes were as follows:

(1) 240 g L^−1^ CuSO_4_ × 5 H_2_O + 60 g L^−1^ H_2_SO_4_; (named as “*electrolyte 1*”);
(2) 240 g L^−1^ CuSO_4_ × 5 H_2_O + 60 g L^−1^ H_2_SO_4_ + 0.124 g L^−1^ NaCl (named as “*electrolyte 2*”);
(3) 240 g L^−1^ CuSO_4_ × 5 H_2_O + 60 g L^−1^ H_2_SO_4_ + 0.124 g L^−1^ NaCl + 1 g L^−1^ polyethylene glycol MW 6000 (named as “*electrolyte 3*”); and
(4) 240 g L^−1^ CuSO_4_ × 5 H_2_O + 60 g L^−1^ H_2_SO_4_ + 0.124 g L^−1^ NaCl + 1 g L^−1^ PEG 6000 (polyethylene glycol) + 0.0015 g L^−1^ MPSA (3–mercapto–1–propanesulfonic acid) (named as “*electrolyte 4*”).

Cold-rolled polycrystalline copper foil (125 μm thick) was used as the cathode in the electrodeposition processes.

The electrolytes were prepared using high-purity water (Millipore, Burlington, MA, USA, 18 MΩ·cm) and p.a. reagents. Depending on the characterization method, the surface areas of the cathodes were either (1.0 × 1.0) or (10.0 × 2.0) cm^2^. The Cu cathodes were firstly mechanically treated with SiC paper (1500 #) and then rinsed in distilled water, etched in 20% H_2_SO_4_ at 50 ± 1 °C, and rinsed again with distilled water. Pure copper was used as the anode in all experiments. The film thickness was checked by a mechanical comparator (Iskra, NP37, “Iskra Avtomatica”, Ljubljana, Slovenia).

### 2.2. Characterization Methods

#### 2.2.1. Surface Characterization of Electrodeposited Cu Films

The morphology of synthesized Cu films was characterized by a scanning electron microscope (SEM; model JEOL JSM-6610LV, JEOL Ltd., Tokyo, Japan).

Topography and roughness of Cu films were investigated on an atomic force microscope (AFM; model Auto Probe CP Research; TM Microscopes–Veeco Instruments, Santa Barbara, CA, USA). Statistical roughness parameters are used to describe the surface morphology from the AFM pictures in a quantitative way. These parameters include the arithmetic average of the absolute roughness (*R*_a_) and the root mean square roughness (*R*_q_) [44]. The height of grains in the form of histograms and bearing ratio curves are obtained from the mean image data plane, combining the software SPMLab (SPMLab NT Ver. 6.0.2., Veeco Instruments, Santa Barbara, CA, USA), Gwyddion (free version), and WsXM 4.0 version Beta 9.3 [45] and a standard Abbott–Firestone curve (DIN4776; STN ISO 13565-2) [46,47,48,49,50]. The scan size was (50 × 50) µm^2^ and the non-contact mode was used.

The crystallite size, the lattice strain, and the texture of Cu films on the Cu cathode were examined using the X-ray diffraction (XRD) method using a RIGAKU Ultima IV diffractometer (Rigaku Co., Ltd., Tokyo, Japan). The Bragg–Brentano geometry with CuKα radiation in a 2*θ* range from 35° to 95° was used. The average crystallite size of Cu films was calculated using the Williamson–Hall and Halder–Wagner methods, while lattice strain was evaluated according to the size–strain plot method [51]. The crystallite size was also determined using the Debye–Scherrer equation [52], and correlation with the Hall–Petch equation [53,54] was performed.

#### 2.2.2. Mechanical Characterization of Electrolytically Produced Cu Films—Microhardness and Adhesion Investigations

**Figure 1:**
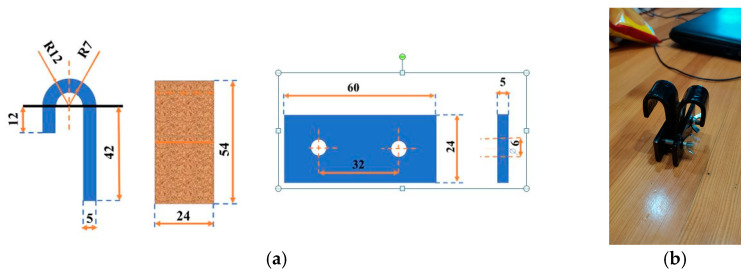
(**a**) Shape and dimensions of the steel structure used as a sample support (**left**) and shape and dimensions of the steel clamp/splint (**right**), and (**b**) the lab-made construction of the machine for the bidirectional bending test.

Three different methods were used to evaluate the adhesion of Cu films:

(1) The microindentation adhesion test (mechanical/non-destructive method) considers a quantitative criterion called “critical reduced depth” or “parameter *b*”, which is obtained by application of the Chen–Gao (C–G) composite hardness model [37,38,39,40,41,42,43]. For the calculation of this criterion, it is necessary to know the intrinsic (real or absolute) hardness of the Cu films. The intrinsic hardness of the film is calculated from the measured or composite hardness of the film by application of an appropriate composite hardness model (CHM). A Vickers microhardness tester “Leitz Kleinert Prufer DURIMET I” (Leitz, Oberkochen, Germany) with applied loads (*P*) between 49 mN and 2.94 N and a dwell time of 25 s was used for the determination of the composite hardness of electrodeposited films. The definition of the composite or the measured hardness of a film is given in the Supplementary Materials.
(2) The bidirectional bending test (mechanical/destructive method) records the number of cycles (NCs) up to the appearance of delamination of the film from the substrate. This adhesion test was conducted using the two-way bending method over a special metal construction shown in Figure 1. The samples are bent manually left and right up to the delamination of the film from the substrate. The moment the film peels off from the substrate is counted as a critical cycle, and the critical cycle number (NC) is noted. The dimensions of the construction of the special bending fixture (Figure 1a) are taken from the literature [7], but the real construction was realized in lab-made conditions (Figure 1b).
(3) The scratch-tape adhesion test is a standard test method for measuring the adhesion of films thinner than 125 μm and belongs to a group of mechanical/destructive methods [8]. This test is qualitative and descriptive, and the test method B type—the cross-cut tape test—was used in this study. For metals and a film thickness thinner than 125 μm, lattice patterns with six slices in each direction were created on the film surface using a crosshatch kit. The size of every grid square was (1.0 × 1.0) mm^2^. Pressure-sensitive tape (3M Deutschland GmbH, Neuss, Germany) was placed over the lattice, and after 90 s, it was quickly removed from the lattice. The film adhesion was estimated on the basis of a contrast observed after delamination of the film from the substrate by an analysis of optical microscopy (OM) images. The films are categorized as follows: 5B (without delamination), 4B (less than 5% delaminated surface), 3B (5–15%), 2B (15–35%), 1B (35–65%), and 0B (greater than 65%) [8,55].

## 3. Results

### 3.1. Morphological, Topographic and Structural Analyses of Electrodeposited Cu Films

#### 3.1.1. Morphological Analysis of Electrodeposited Cu Films

**Figure 2:**
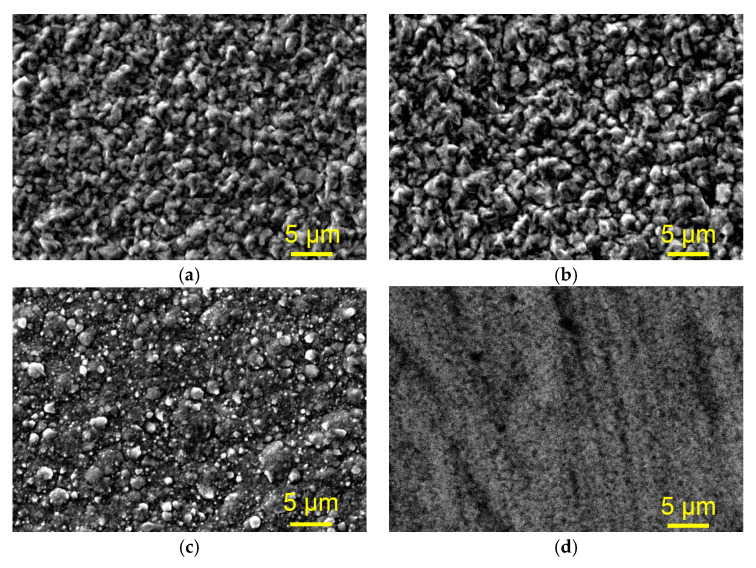
SEM images of the copper films electrodeposited on the copper substrate from the basic sulfate electrolyte without/with the addition of additives at a current density of 60 mA cm^−2^: (**a**) *electrolyte 1*, (**b**) *electrolyte 2*, (**c**) *electrolyte 3*, and (**d**) *electrolyte 4*. The film thickness: 10 µm. Magnification: 3000×.

Figure 2 shows SEM images of the morphology of electrodeposited Cu films on the copper substrate obtained from the basic sulfate electrolyte (*electrolyte 1*, Figure 2a), the basic electrolyte with the addition of chloride ions (*electrolyte 2*, Figure 2b), the basic electrolyte with the addition of both chloride ions and polyethylene glycol (PEG 6000) (*electrolyte 3*, Figure 2c), and the basic electrolyte with the addition of all three additives (*electrolyte 4*, Figure 2d). It can be observed from Figure 2 that the presence of additives has a strong effect on the morphology of electrodeposited Cu films. The morphologies of the films obtained from *electrolyte 1* and *electrolyte 2* are fine-grained and very similar to each other at the macro level (Figure 2a,b). The addition of PEG 6000 makes the Cu film non-uniform and coarser, with various grain sizes (Figure 2c). The Cu film obtained in the presence of all three additives was very smooth (Figure 2d), and this combination of additives gave the film a mirror-bright appearance [15]. As seen from Figure 2d, the addition of PEG 6000 + chloride ions + MPSA (*electrolyte 4*) reduced the grain size in the film up to the order of nanometer dimensions, causing a loss of clear difference among grains in the film.

#### 3.1.2. Topographic Analysis of Electrodeposited Cu Films

**Figure 3:**
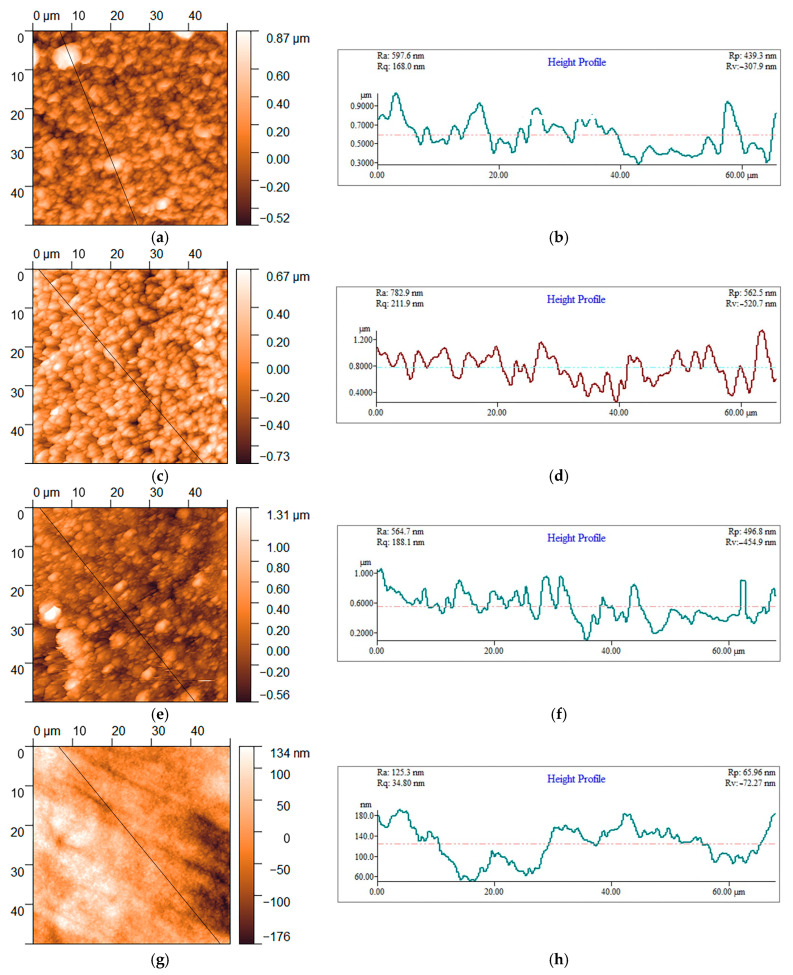
The surface topography (**a**,**c**,**e**,**g**) and the line section analysis (**b**,**d**,**f**,**h**) of the 10 µm thick Cu films produced by the DC/MS mode at a current density of 60 mA·cm^−2^ from (**a**,**b**) *electrolyte 1*, (**c**,**d**) *electrolyte 2*, (**e**,**f**) *electrolyte 3*, and (**g**,**h**) *electrolyte 4*.

With the aim of a quantitative analysis of formed Cu films, further characterization of the Cu films was performed by applying the AFM technique and accompanying software (Gwyddion Ver. 2.65). Figure 3 shows the 2D (two dimensional) AFM images and corresponding line section profiles of Cu films obtained without and with additives in the basic sulfate electrolyte. The roughness parameters, such as the arithmetic average of the absolute roughness (*R*_a_) and the root mean square roughness (*R*_q_), for the Cu films are given in Table 1.

**Figure 4:**
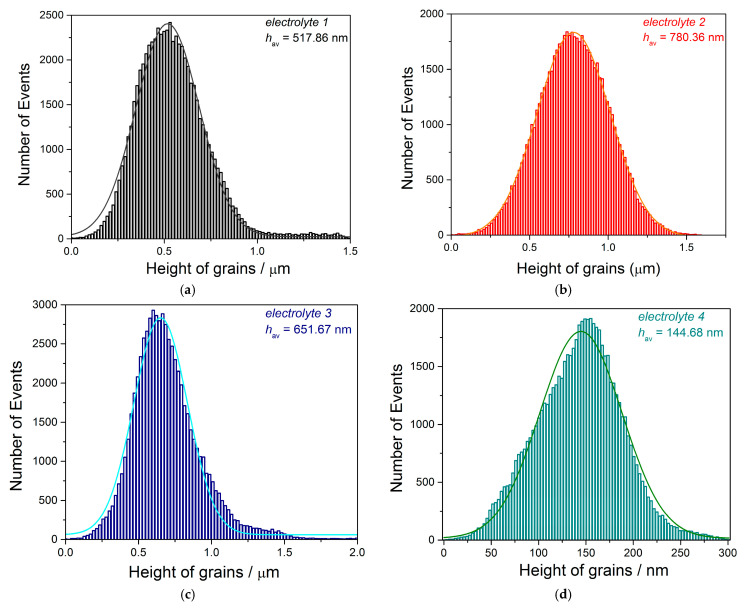
The histograms for 10 µm thick Cu films obtained on the Cu cathode in the DC/MS mode from (**a**) *electrolyte 1*, (**b**) *electrolyte 2*, (**c**) *electrolyte 3*, and (**d**) *electrolyte 4*.

The AFM histograms of the topography of Cu films produced from all four electrolytes were derived from the AFM images shown in Figure 3 and are given in Figure 4. Based on the histogram analyses, the average height of grains (*h*_av_) is obtained by application of a Gaussian distribution function, and the obtained values are added to Table 1. Analysis of the histograms showed that the most uniform Cu film with the smallest grain size was obtained with the combination of all three additives (Figure 4d).

**Figure 5:**
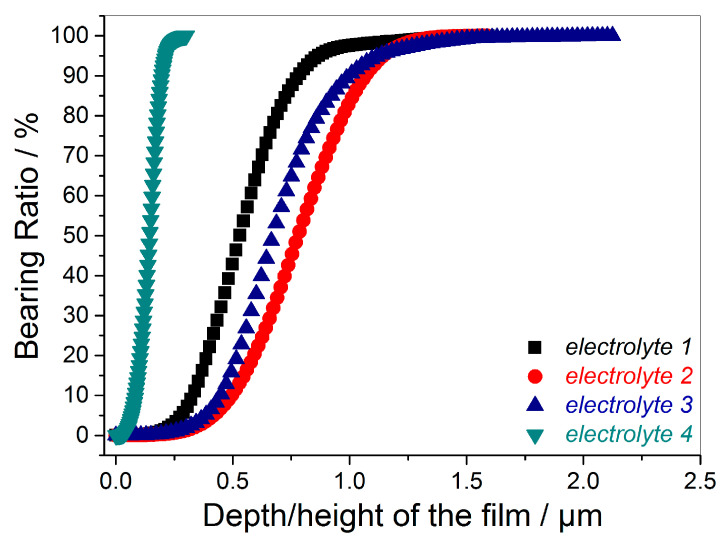
The Abbott–Firestone curves for 10 µm thick copper films obtained in the DC/MS regime from different electrolytes (*electrolyte 1*, *electrolyte 2*, *electrolyte 3*, and *electrolyte 4*).

Additional information about the topography of the Cu films is obtained by the analysis of the so-called bearing ratio curves, also known as the Abbott–Firestone curves [46,47,48,56]. These curves, derived from the AFM images for the given films, are presented in Figure 5. The main parameters that can be extracted from the Abbott–Firestone curves are as follows: *R*_pk_—reduced peak height; *R*_k_—core roughness depth; and *R*_vk_—reduced valley depth (ISO 13565 standard) [57]. It can be noted that the Abbott–Firestone curves separately estimate the peak height and the valley depth of the film above and below arbitrary plane. The values of these parameters are added to Table 1, while the method of their determination is presented in the Supplementary Materials (see Figure S1).

It can be seen from Figure 5 that the Abbott–Firestone values for the Cu film electrodeposited from *electrolyte 4* approach the maximum rapidly, whereas for other Cu films these values approach more slowly. Analysis of the data from Table 1 confirmed that the mirror-bright film had a more uniform topography than other films, in which roughness was deeply in the micro range.

#### 3.1.3. Structural Analysis of Electrodeposited Cu Films

**Figure 6:**
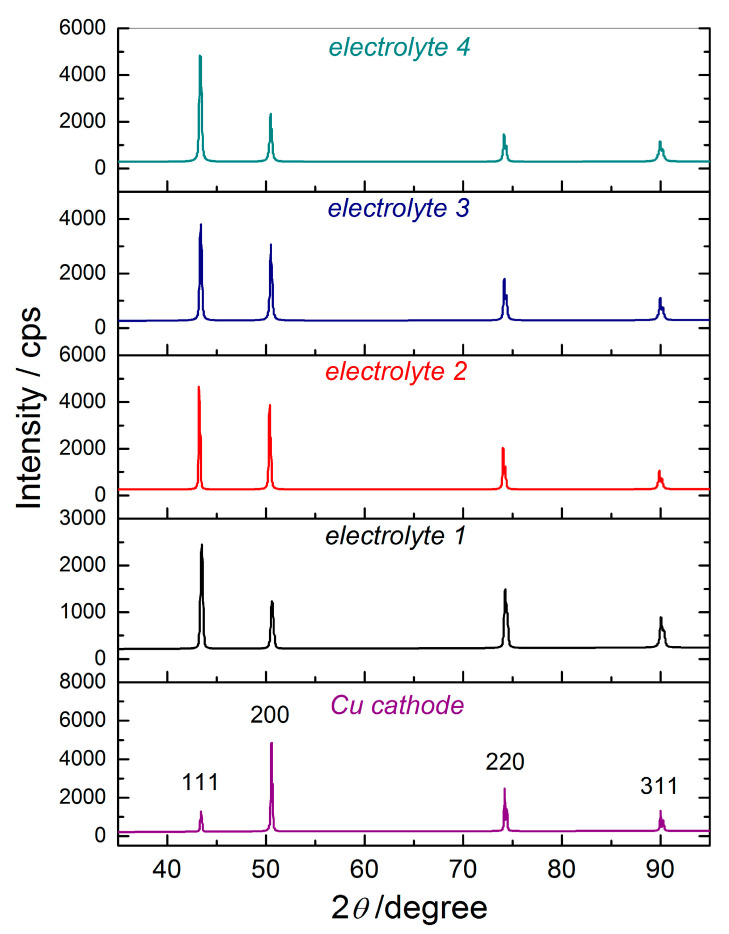
The X-ray diffraction (XRD) patterns obtained for the Cu cathode and the Cu films electrodeposited from *electrolytes 1*, *2*, *3*, and *4*.

The XRD patterns of the Cu cathode and the Cu films obtained from various electrolytes are shown in Figure 6. At first sight, a strong structural modification of the Cu cathode by the electrodeposition process is observed. Comparison of the diffraction peaks observed at 2*θ* angles of 43.3°, 50.4°, 74.1°, and 89.9°, which belong to the main crystal planes of Cu ((111), (200), (220), and (311)) with the standard for Cu (JCPDS No. 04-0836), confirmed that pure Cu was electrodeposited from the electrolytes without and with different additives.

The strongest diffraction peak corresponding to the (200) crystal plane was observed in the Cu cathode, while that corresponding to the (111) crystal plane was characteristic of the diffractograms belonging to the electrodeposited Cu films. Simultaneously, the mutual ratio of (200), (220), and (311) diffraction peaks in the films depended on the composition of the electrolyte. Regarding the electrodeposited films, it is clear that the addition of additives favoured crystal growth in the (200) crystal plane relative to the (220) and (311) crystal planes.

### 3.2. Mechanical Characterization of Electrodeposited Cu Films—Analysis of Hardness and Adhesion Characteristics

The estimation of the adhesion properties of the Cu films by the use of the microindentation method is based on the application of the Chen–Gao composite hardness model (C–G CHM) [37,38,39,40,41,42,43]. This theoretical model defines a “critical reduced depth” or “adhesion parameter”, *b*, which in a physical sense represents the ratio between the radius of the plastic zone beneath the indentation and the indentation depth [37,38,39,40,41,42,43]. The adhesion parameter, *b*, is defined by Equation (1) [40,41]:

(1) $$b=\frac{7(m+1)(H_{f}-H_{s})}{m\Delta H}\frac{\delta }{d}$$

where *H*_f_ is the intrinsic hardness of the film, *H*_s_ is the cathode (substrate) hardness, *m* is the power index, *δ* is the film thickness, and *d* is the diagonal size measured in an indentation at the surface area of the film. Δ*H* represents the difference between substrate hardness and measured hardness by Vickers microindentation (so called the composite hardness of a film), *H*_c_, i.e., Δ*H* = *H*_s_ − *H*_c_.

During the determination of the hardness of metallic films by the microindentation method, the influence of substrate hardness must be taken into account, especially where thin films are concerned, which is the case in this study. The measured (composite) hardness is a complex quantity, including contributions from both the hardness of the film (*H*_f_) and the hardness of the substrate (*H*_s_) [37,38,39,40,41,42,43,58,59,60,61]. For the determination of the intrinsic hardness of Cu films, it is necessary to know the value of the absolute hardness of the substrate (cathode). In our case, the absolute hardness of the substrate was determined by the Proportional Resistance Model (PSR) mode [62], and the calculated value for the hardness of the Cu substrate was 0.56 GPa [63].

The intrinsic hardness of films is commonly determined by the application of a suitable composite hardness model (CHM), where the choice of model depends on the film/substrate hardness ratio [61]. Cu films electrodeposited on Cu cathodes belong to the “hard film on soft substrate” composite hardness type, and for this system, the Korsunsky (K) model [64,65] proved to be very successful in the determination of the intrinsic hardness of the Cu films [66].

According to the K model, the dependence of the composite hardness, *H*_c,_ on the diagonal size, *d*, is given by Equation (2) [64,65]:

(2) $$H_{c}=H_{s}+\left[\frac{1}{1+k`\cdot \left(d^{2}/\delta \right)}\right]\cdot \left(H_{f}-H_{s}\right);k`=\frac{k}{49\cdot \delta }$$

where *k* and *k*′ are parameters obtained by fitting and related to the composite hardness response mode.

**Figure 7:**
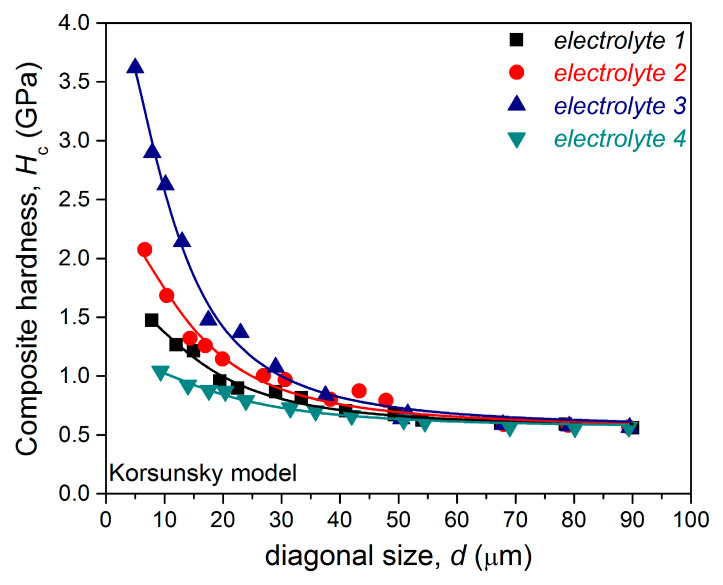
The dependencies of the measured composite hardness, *H*_c,_ on the diagonal size, *d*, and fitted dependencies obtained for the Cu films electrodeposited on the Cu cathode from *electrolyte 1*, *electrolyte 2*, *electrolyte 3*, and *electrolyte 4*.

The experimentally obtained dependencies of *H*_c_ on *d*, with included fitted dependencies for the Cu films electrodeposited on the Cu cathode from all four electrolytes, are presented in Figure 7.

All curves feature a downward trend in the composite hardness values, with an increase in the diagonal size, confirming that Cu/Cu films belong to the “hard film on soft substrate” composite hardness system type. For a diagonal size of around 50 µm, the composite hardness reaches a value of around 0.56 GPa, corresponding to the hardness of the Cu substrate. This means that the contribution of the film hardness to the composite hardness tends to zero. For a diagonal size less than 10 µm, the influence of the film hardness on the measured composite hardness becomes dominant.

The calculated values of the film hardness (*H*_f_), fitting parameters, and errors for the analyzed Cu films are given in Table 2.

**Figure 8:**
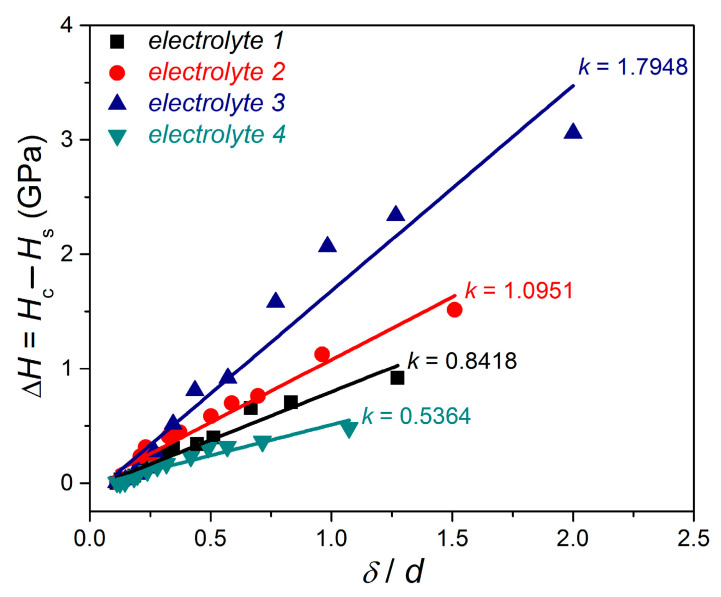
Hardness difference vs. ratio between the film thickness and the indentation diagonal for copper films electrodeposited from *electrolytes 1*, *2*, *3,* and *4*.

In the next step, to estimate the adhesion parameter, *b,* according to the C–G model, the dependencies of Δ*H* on *δ*/*d* are determined (see Equation (1)), and for the given films they are shown in Figure 8. The value of parameter m in Equation (1) depends on the substrate/film hardness ratio, and for the “hard film on soft substrate” composite hardness system, the value of parameter *m* is 1.2 [37,38,39,40,41,42,43]. A linear fit of all data was also performed and is included in this Figure 8. The calculated values of slope *k* are reported in the same figures and were used to calculate values of adhesion parameter *b*. The values of the calculated adhesion parameters were 14.439, 19.347, 22.267, and 11.376 for *electrolytes 1*, *2*, *3* and *4*, respectively, and they are given in Table 3. It is clear that the largest adhesion parameter, and hence the best adhesion, was shown by the Cu film obtained from *electrolyte 3,* i.e., the film electrodeposited from the electrolyte with added chloride ions and PEG 6000. On the other hand, the weakest adhesion was shown by the mirror-bright Cu film obtained with all three additives. The sequence of the change in adhesion properties of Cu films was as follows: *electrolyte 3* > *electrolyte 2* > *electrolyte 1* > *electrolyte 4*.

**Figure 9:**
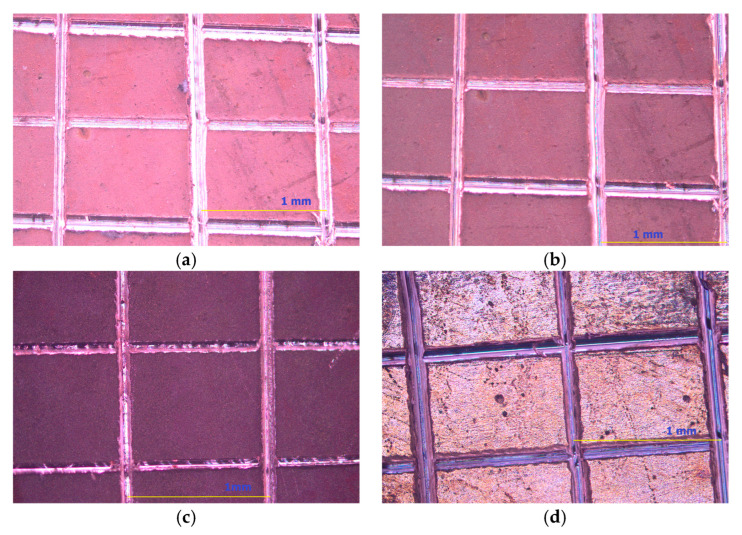
The photographs of grids performed according to the ASTM D3359 standard obtained by optical microscope for the copper films electrodeposited on the Cu substrate from (**a**) *electrolyte 1*, (**b**) *electrolyte 2*, (**c**) *electrolyte 3*, and (**d**) *electrolyte 4*.

To verify the results of the adhesion estimation of the Cu films calculated using the C–G CHM, two additional experimental methods were applied: (a) a bidirectional bending test and (b) scratch-tape adhesion test. The values of the critical cycle number (NC) obtained by the bidirectional bending test and test method B obtained by the scratch-tape adhesion method for given Cu films are added to Table 3. The appearance of Cu films after the scratch-tape test is shown in Figure 9.

The following trend of the change in adhesion properties of the Cu films was observed by application of the bidirectional bending test: *electrolyte 3* > *electrolyte 2* > *electrolyte 1* > *electrolyte 4*. Also, it is shown that there was no difference in the adhesion category when the scratch-tape adhesion test was applied and that all Cu films showed the strongest adhesion category (5B), i.e., delamination of the films from the cathodes was not observed.

Analysis of the data given in Table 3 showed a good agreement in the estimation of the adhesion properties of the Cu films by application of the theoretical C–G CHM with pure experimentally obtained values. This clearly shows that composite hardness models such as the Chen–Gao (C–G) model can be successfully applied in the estimation of the adhesion of the films in the absence of purely experimental methods.

## 4. Discussion of the Presented Results

The adhesion of thin Cu films galvanostatically electrodeposited on a Cu cathode from electrolytes without and with different additives has been considered in this study. The Cu films were formed by the ED process from a basic sulfate electrolyte (*electrolyte 1*), an electrolyte with added chloride ions (*electrolyte 2*), an electrolyte to which chloride ions and PEG 6000 were added as additives (*electrolyte 3*), and an electrolyte with a combination of additives, such as chloride ions, PEG 6000, and 3–mercapto–1–propanesulfonic acid, which gave a mirror-like appearance to the Cu film (*electrolyte 4*). The ED process performed at the same current density caused the formation of films with different morphologies, starting from fine-grained films of different degrees of roughness obtained from *electrolytes 1, 2,* and *3* to the film of nano-sized dimensions obtained from *electrolyte 4*.

Strong structural modification of the initial cathode surface was observed. The Cu crystallites were predominately oriented in the (111) crystal plane in all types of Cu films, clearly pointing out the Cu ED process favors crystal growth in the (111) crystal plane, which is the crystal plane with the lowest surface energy in the face-centered cubic structure of Cu [67].

The composition of the electrolytes affected not only the morphological characteristics of the Cu films, but also their mechanical characteristics. The highest hardness and the best adhesion were observed for the Cu film electrodeposited from the *electrolyte 3*, i.e., with added chloride ions and PEG 6000. The strong correlation between the morphological and mechanical characteristics of Cu films can be explained and discussed as follows: The addition of chloride ions to the simple sulfate electrolyte did not have any significant effect on the morphology of the film. Both films are fine-grained, with the grains at the micro scale and clear boundary among them (Figure 2a,b). However, an increase in roughness of the Cu film was observed with the presence of chloride ions in the electrolyte from *R*_a_ = 153.0 nm for the film obtained from the basic sulfate electrolyte to *R*_a_ = 180.0 nm for that obtained with added chloride ions. The increase in the hardness of the film deposited in the presence of chloride ions compared to the additive-free electrolyte can be attributed to this change in roughness of the films. Namely, according to the Hall−Petch effect [68], films with more grains and, consequently, with a larger number of boundaries among them exhibit greater hardness because each grain boundary acts as a barrier when the indenter penetrates in the film, decreasing the diagonal size during microindentation [69]. Additionally, an increase in the hardness of a film might result from the electrolyte particles at the grain borders blocking dislocation motion and grain boundary sliding [70].

The largest hardness and the best adhesion were exhibited by the Cu film electrodeposited from the electrolyte containing both chloride ions and PEG 6000. The addition of these two additives had a significant impact on the surface morphology of the Cu film. This Cu film was finer-grained, with a larger number of boundaries among grains than in those obtained without and with the addition of only chloride ions, but with spherical grains, which increased its roughness (Figure 2c). This morphology of the film can be explained by the synergistic effect of the chloride ions and PEG 6000, where these two additives adsorb on the cathode surface as a copper chloride complex, with the polymer functioning as a ligand [71]. Kelly and West [72,73] found that when PEG and chloride ions are present in the electrolyte, mono-layer PEG rapidly collapses into spherical aggregates, as shown in Figure 2c. The present spherical forms behave like elastic balls, so that during the microindentation test they absorb parts of the penetration energy of the indenter. The improvement in adhesion properties of the Cu film in the presence of chloride ions and PEG 6000 can also be attributed to characteristics of PEG molecules. It is a polymer with hydrophilic and antistatic properties, and as such it can enhance the adhesion properties of metal films by an improvement in surface compatibility and by a reduction in biofouling [74]. PEG has a relatively high surface energy, which contributes to its hydrophilic nature and ability to resist adsorption, making PEG very useful for adhesion enhancement in film technology [74].

Finally, the lowest hardness and the worst adhesion are observed for the mirror-bright Cu film obtained with a combination of all three additives (*electrolyte 4*). This film was very smooth, with the grains at the nano scale, and without a clear boundary among them. During microindentation, the film behaved as though constructed from a smaller number of larger grains, making the diagonal size on the film surface larger than in the case of fine-grained films [58,75]. Softer copper films like that made from *electrolyte 4* have a very high pile-up effect along the border of the indent [58]. However, the pile-up effect is not observed around the indents for the Cu films obtained from *electrolytes 1*, *2,* and *3* (see Supplementary, Figure S2). For these films, plastic deformation of the film beneath the indenter is the dominant process. This explains the greater hardness of the Cu films made from *electrolyte 1*, *2,* and *3* over that made from *electrolyte 4*. The nano size of grains in mirror-bright films is below the critical value for validity of the Hall−Petch equation, and for this type of film, the inverse Hall–Petch equation starts to hold, causing lower film hardness than in those with grains at the micro level [69]. This is also in accordance with the C–G CHM, predicting that softer films show worse adhesion than harder films [37,38,39].

The lattice strain, *ε,* is another parameter affecting mechanical properties, and, hence, the adhesion of metal films. This parameter can be calculated from the XRD data according to the Williamson–Hall (W–H) and Halder–Wagner (H–W) methods [51,52,53,54], and these values calculated according to the W–H method were 0.05, 0.08, 0.01, and 0.09% for the Cu films electrodeposited from *electrolytes 1*, *2*, *3,* and *4*, respectively. Similar values were also obtained using the H–W method, and the calculated values were 0.01, 0.07, 0.01, and 0.08% for the Cu films electrodeposited from *electrolytes 1*, *2*, *3,* and *4*, respectively. The values of crystallite size used for the calculation of the lattice strain values, but also those obtained by the application of the Debye–Scherrer (D–S) method, are given in Table S1 in the Supplementary Materials.

The highest lattice strain was extracted for the nanocrystalline mirror-bright Cu film electrodeposited from *electrolyte 4*, while the lowest value was observed for the microcrystalline Cu film electrodeposited from *electrolyte 3*. Hence, it is clear that the stretching of the crystallographic lattice is associated with the reduction in adhesion properties of the film. The trend of change in these values was in line with the change in the hardness and the adhesion strength of prepared Cu films, proving the strong correlation between the structural and mechanical properties of the Cu films. The effect of the combination of all three additives on the weakening of the adhesion of the copper film electrodeposited from *electrolyte 4* relative to *electrolytes 1*, *2,* and *3* can also be attributed to the reduction in the pinholes in the films and to the reduction in the film tension [76].

The increase in the adhesion of prepared Cu films was also accompanied by the lowering of the residual stress in the film [77]. Namely, monotonous growth tensile/compressive residual stresses were observed in the films obtained with the addition of leveling/brightening additives, where it is assumed that the entrapped impurities from the additives represent the source of the residual stress. To put it another way, the integration of entrapped sulfur from MPSA and entrapped hydrogen from PEG is responsible for the compressive and tensile stresses for the Cu film electrodeposited from *electrolyte 4*. This fact explains the lowest value of the NC parameter obtained after the bending test (Table 3). The values of residual stresses in Cu films depend on the residual stress of each crystal plane and it is related to material parameters, such as Young′s modulus (for pure copper it is *E* = 1.078 × 10^10^ N m^−2^), Poisson’s ratio (0.35), and the crystal plane spacing of the standard card [78]. The increase in the intensity of the diffraction peak (Figure 6) indicates that Cu films formed in baths with additives had increased residual stress compared with the Cu film obtained from the electrolyte without additives. Also, the peak corresponding to the (111) crystal plane for the Cu film electrodeposited from *electrolyte 4* shifted to the right side. This is attributed to the reduction in crystal plane spacing due to the induction of residual stress in the thin film [78,79].

The relationship between the morphology and adhesion of the Cu films can also be explained from an electrocrystallization point of view, as follows: the addition of various additives to an electrolyte usually increases the cathodic polarization [22,80]. This means that the increase in the overpotential as a potential response occurs when the ED process performs at the same current density for the electrolyte with additives relative to that without additives. The nucleation rate strongly increases with the increase in the overpotential [15], with the consequence that a larger number of nuclei are formed at the cathode surface in the initial stage of electrodeposition. Since the ED process performs at the same current density, the same electrodeposited charge is then distributed on the crystal growth of a larger number of initially formed nuclei, leading to the formation of a film with a smaller grain size as the final surface morphology. Finally, the larger number of initially formed nuclei on the cathode surface causes better adhesion of the freshly formed film with the cathode.

In addition, there is a strong correlation between the morphological and mechanical characteristics of electrodeposited thin Cu films on the Cu substrate. Increasing the surface roughness of the film proved to be beneficial for improving the adhesion of the film to the substrate. The morphology and hence the roughness of the film can be regulated by the composition of the electrolyte, as considered in this study, but also via the settings of current density used in the ED process, the choice of regime of electrodeposition (constant or pulse reverse current (PRC) regimes), and the selection of parameters of electrodeposition, such as temperature, deposition time, electrolyte stirring, etc. In this way, the variation in these ED parameters and regimes enables optimizing the mechanical characteristics of Cu films.

## 5. Conclusions

The adhesion properties of copper thin films obtained by the galvanostatic regime of electrodeposition on Cu cathodes were evaluated. Cu films were electrodeposited from electrolytes of different compositions, including a basic sulfate electrolyte (*electrolyte 1*) and electrolytes with the following additives: chloride ions (*electrolyte 2*), chloride ions and PEG 6000 (*electrolyte 3*), and chloride ions, PEG 6000, and 3–mercapto–1–propanesulfonic acid (*electrolyte 4*). The produced films were characterized by scanning electron microscopy and atomic force microscopy (morphology) and by X-ray diffraction (structure) techniques. The adhesion properties of the films were evaluated by use of the Chen–Gao (C–G) composite hardness model (CHM) based on results obtained by Vickers microindentation, bidirectional bending test, and scratch-tape adhesion test. The hardness values of Cu films necessary for the estimation of adhesion by the C–G CHM were determined using the Korsunsky CHM. Based on obtained results, the following conclusions can be drawn:

- Fine-grained films with micro-sized grains were formed from *electrolytes 1, 2,* and *3*. The roughness of the films changed as follows: *electrolyte 3* > *electrolyte 2* > *electrolyte 1*. The Cu film obtained from *electrolyte 4* had nano-sized dimensions with a mirror-bright appearance.
- The trend of change in hardness and adhesion of the films was as follows: *electrolyte 3* > *electrolyte 2* > *electrolyte 1* > *electrolyte 4*.
- The best adhesion was exhibited by the film with the largest roughness, produced from *electrolyte 3*, and the worst adhesion was exhibited by the mirror-bright film produced from *electrolyte 4*.
- Good agreement in the estimation of film adhesion was achieved by application of combined theoretical and experimental (C–G CHM using Vickers microindentation results) methods and pure experimental (bidirectional bending test and scratch-tape adhesion test) methods.
- The different adhesion of the produced Cu films was explained and discussed by analysis of phenomena occurring on boundaries among grains (mechanical approach) and the effect of additives on the ED process (electrochemical approach).

## Figures and Tables

**Table 1:** The roughness parameters, *R*_a_—the arithmetic average of the absolute roughness; *R*_q_—the root mean square roughness; *R*_pk_—reduced peak height; *R*_k_—core roughness depth; *R*_vk_—reduced valley depth, and an average value of grain size (*h*_av_) for 10 µm thick copper films obtained in the DC/MS regime on Cu cathodes from different electrolytes.

Electrolyte	*R*_a_ (nm)	*R*_q_ (nm)	*R*_pk_ (nm)	*R*_k_ (nm)	*R*_vk_ (nm)	*h*_av_ (nm)
1	153.0	199.9	307.1	446.1	837.0	517.86
2	180.0	223.7	490.1	587.0	511.4	780.36
3	194.3	257.1	422.3	505.6	1197.1	651.67
4	46.09	58.97	91.0	108.1	101.7	144.68

**Table 2:** Results of the real hardness measurements for the copper films electrodeposited on the copper substrate type from *electrolytes 1*, *2*, *3,* and *4* according to the Korsunsky (K) composite hardness model. The errors are *SE*—Standard Error; *RSE*—Relative Standard Error.

Electrolyte	Korsunsky Composite Hardness Model		
	Fitting Parameter (*k*′) and Errors (*SE* and *RSE*)		Hardness of the Film
	*k*′ (*SE*)	*RSE*	*H*_f_ (GPa)
1	19.84278 (2.48141)	0.98478	1.7021
2	23.58663 (3.81798)	0.97084	2.3156
3	39.91077 (3.48558)	0.99406	3.2161
4	13.15879 (1.68219)	0.98335	1.1475

**Table 3:** The values of the calculated adhesion parameter, *b*, the critical cycle number, NC, and the values of test method B.

Electrolyte	Adhesion Parameter, *b*	The Critical Cycle Number, NC	The Value of Test Method B
1	14.439	21.5	5B
2	19.347	24	5B
3	22.267	40	5B
4	11.376	13	5B

## Data Availability

The data presented in this study are available on request from the corresponding authors due to reasonable request.

## Supplementary Materials

The following supporting information can be downloaded at https://www.mdpi.com/article/10.3390/ma18112480/s1, Figure S1: The Abbott–Firestone curve the Cu film electrodeposited from *electrolyte 1*.; Figure S2: Optical microscopy images obtained after Vickers indentation with an applied load of 0.9806 N on the Cu films electrodeposited from: (a) *electolyte 1*, (b) *electolyte 2*, (c) *electrolyte 3* (no pile-up effect), and (d) *electrolyte 4* (pile-up effect).; Table S1: The values of calculated crystallite size calculated from XRD peak broadening for the Cu films electrodeposited from *electrolytes 1*, *2*, *3*, and *4* at a current density of 60 mA·cm^−2^. References [56,81] are cited in the Supplementary Materials.
