# Supplementary material for: Evaluation of Adhesion Properties of Electrodeposited Copper Thin Films: Theoretical and Experimental Approach
Source: Materials (Basel). 2025 May 25;18(11):2480. doi: 10.3390/ma18112480 (PMC12156451; doi:10.3390/ma18112480)
Supplement: Supplementary file 1 [file materials-18-02480-s001.zip › materials-3635707-supplementary.pdf]

Supplementary Material

1. Definition of the composite (measured) hardness

The composite or measured hardness (H_c) including a contribution of the substrate hardness (i.e. cathode) is given by Eq. (S1) [R1]:

$$H_c = \frac{1.8544 \cdot P}{d^2} \tag{S1}$$

where H_c (in Pa) is the measured (or the composite hardness), P (in N) is the applied load, and d is size of a diagonal (in m) in the film obtained under the applied load.

2. Bearing Ratio (Abbott-Firestone) Curve

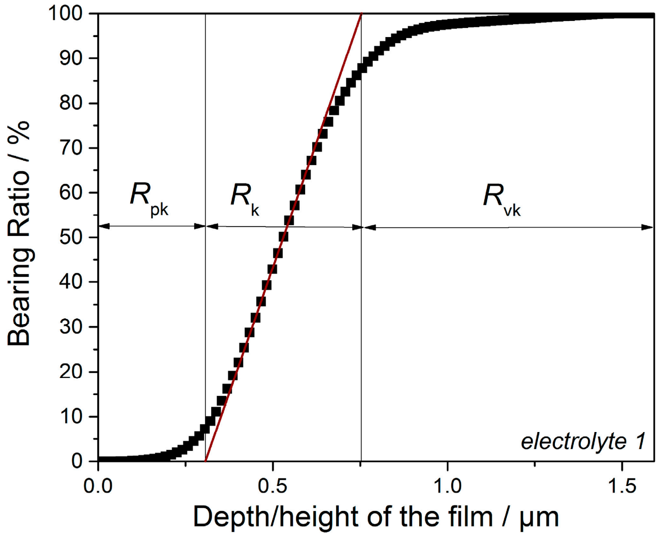

Figure S1. The Abbott–Firestone curve the Cu film electrodeposited from *electrolyte 1*.

The parameters: R_{pk} – reduced peak height, R_k – core roughness depth, and R_{vk} – reduced valley depth [R2].

3. The appearing of the pile-up effect at the surface area of the film along the border of the indent

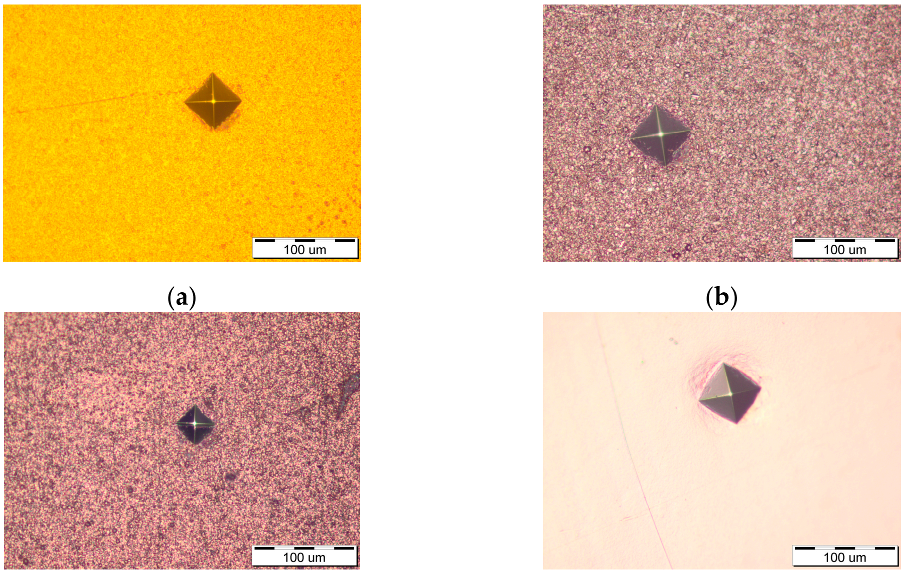

(**a**) (**b**) (**c**) (**d**)

Figure S2. Optical microscopy images obtained after Vickers indentation with an applied load of 0.9806 N on the Cu films electrodeposited from: a) *electolyte 1*, b) *electolyte 2*, *c) electrolyte 3* (no pile-up effect), and d) *electrolyte 4* (pile-up effect).

4. The values of crystallites size used for a calculation of the lattice strain obtained by an application of Williamson–Hall (W–H), Halder–Wagner (H–W) and Debye–Scherrer (D–S) methods

Table S1. The values of calculated crystallite size calculated from XRD peak broadening for the Cu films electrodeposited from *electrolytes 1*, *2*, *3*, and *4* at a current density of 60 mA·cm^{-2}.

**Methods**	**W–H**		**H–W**		**D–S**
**The type of electrolyte**	***D / nm***	***ε / %***	***D / nm***	***ε / %***	***D / nm***
electrolyte 1	27.2	0.05	31.5	0.01	60.3
electrolyte 2	113.9	0.08	76.3	0.07	75.2
electrolyte 3	41.1	0.01	41.5	0.01	35.7
electrolyte 4	55.7	0.09	37.3	0.08	45.5

References:

R1. Mladenović, I.O.; Lamovec, J.S.; Vasiljević Radović, D.G; Vasilić, R.; Radojević, V.J.; Nikolić, N.D. Morphology, Structure and Mechanical Properties of Copper Coatings Electrodeposited by Pulsating Current (PC) Regime on Si(111). *Metals* **2020**, *10*, 488. https://doi.org/10.3390/met10040488.

R2. Hamdi, A.; Merghache, S.M.; Aliouane, T. Effect of cutting variables on bearing area curve parameters (BAC-P) during hard turning process. *Arch. Mech. Eng.* **2020**, 67, 73–95. https://doi.org/10.24425/ame.2020.131684.
